# Grocott Methenamine Silver Staining Is the Optimal Approach to Histological Diagnosis of Pulmonary Cryptococcosis

**DOI:** 10.3389/fmicb.2022.885511

**Published:** 2022-04-29

**Authors:** Suijing Wang, Jieyi Lai, Ruibin Wu, Lihong Zhang, Mayan Huang, Yongbo Xiao, Xinke Zhang, Jiewei Chen

**Affiliations:** ^1^State Key Laboratory of Oncology in South China, Collaborative Innovation Center for Cancer Medicine, Sun Yat-sen University Cancer Center, Guangzhou, China; ^2^Department of Pathology, Sun Yat-sen University Cancer Center, Guangzhou, China; ^3^Department of Pathology, Shunde Hospital, Southern Medical University, Foshan, China

**Keywords:** Grocott methenamine silver, periodic acid-Schiff, Alcian blue, histology, pulmonary cryptococcosis

## Abstract

**Background:**

Histological staining methods for *Cryptococcus* identification vary in accuracy. This study aimed to investigate the clinical value of Grocott methenamine silver (GMS), periodic acid-Schiff (PAS), and Alcian blue (AB) staining in the diagnosis of pulmonary cryptococcosis (PC).

**Methods:**

From April 2004 to June 2021, the clinical and pathological data of 152 patients with PC were collected from the Department of Pathology, Sun Yat-sen University Cancer Center. The sensitivity and identifiability of GMS, PAS, and AB staining for histological diagnosis were systematically evaluated using statistical methods combined with the microscopic characteristics of PC cases.

**Results:**

Statistical analysis showed that the detection rates of GMS, PAS, and AB staining were 100.0% (152/152), 94.7% (144/152), and 81.6% (124/152), respectively. McNemar’s test showed that the sensitivity of GMS was significantly higher than those of PAS (*P* = 0.008) and AB stains (*P* < 0.001). Both PAS and AB stains had obvious non-specific staining, which interfered with the detection of *Cryptococcus*, and increased diagnostic difficulties. In contrast, in GMS staining, *Cryptococcus* spores were prominent with a clean background and were clearly observed at low or medium power magnification, with the identifiability significantly better than those of PAS or AB staining.

**Conclusion:**

GMS staining had sensitivity up to 100%, and identifiability that was better than those of PAS and AB staining. GMS is the best method for histological diagnosis of PC.

## Introduction

*Cryptococcus* is a yeast surrounded by capsules that is widely distributed worldwide. It is often found in pigeon dung, soil, and rotten wood, including *Cryptococcus neoformans* and *Cryptococcus gattii* (Maziarz and Perfect, 2016). Respiratory inhalation is the main mechanism of cryptococcal invasion. Pulmonary cryptococcosis (PC) is a subacute or chronic pulmonary mycosis caused by inhalation of *Cryptococcus* spores. Recently, the incidence of PC has been increasing rapidly (Galanis et al., 2010). The clinical manifestations of PC are atypical, and most patients may be asymptomatic, while some patients present with cough, expectoration, chest tightness, chest pain, or even acute respiratory distress syndrome (Lin et al., 2021). The most common radiological features of PC are single or multiple nodules or mass shadows (Zhang et al., 2020). Due to the lack of specificity in imaging and clinical manifestations of PC, PC is easily missed or misdiagnosed as tuberculosis, lung cancer, or metastasis. If not diagnosed and treated in time, *Cryptococcus* may spread to the central nervous system, leading to cryptococcal meningitis, which causes approximately 181,100 deaths annually (Rajasingham et al., 2017; Setianingrum et al., 2019). Accurate laboratory tests are required for the diagnosis and treatment of PC. At present, histopathological detection of *Cryptococcus* remains the gold standard for the diagnosis of PC. Staining of tissues can play an important role in the timely and accurate diagnosis of *Cryptococcus* infection.

Diagnostic methods for PC include serology, histopathology, fungal culture, and molecular detection. Although the serum methodology is simple, serum cryptococcal polysaccharide antigen testing is usually negative for isolated PC and cryptococcosis with a capsule defect (Ruan et al., 2017; Zhou et al., 2018). The fungal culture process is complex and time-consuming; it is easy to contaminate and yield false-negative results (Zhou et al., 2018). Molecular detection methods are emerging; however, they remain more expensive than other test types, so they are rarely used in laboratories. However, histopathology allows to directly observe the fungal structure, allowing for rapid and intuitive diagnosis. Present methods for PC histological diagnosis include Grocott methenamine silver (GMS), periodic acid-Schiff (PAS), and Alcian blue (AB) staining. However, the sensitivities of these methods differ. No previous study has examined the sensitivities of these methods. In this study, we aimed to identify the most sensitive and identifiable method for the clinicopathological diagnosis of PC, helping establish an evidence base for the selection of diagnostic methods in this context.

## Materials and Methods

### Patient Specimens

All samples were obtained from the Department of Pathology, Sun Yat-sen University Cancer Center (April 2004 to June 2021). All enrolled patients had a confirmed PC diagnosis. The diagnostic criteria for the included PC patients were: sections of lung tissue were stained with GMS, PAS, and AB, and at least one staining was positive for *Cryptococcus*. Hematoxylin and eosin (H&E), GMS, PAS, and AB staining sections were collected from the archives of all enrolled patients, and the clinical data were extracted from medical records. The Institutional Review Board of Sun Yat-sen University Cancer Center approved this study (B2021-452-01).

### Routine and Special Staining

The accessed PC tissue staining sections (*n* = 152) were prepared by trained technicians from the histology laboratory using standardized staining procedures of H&E, GMS, PAS, and AB. All sections were 4-μm thick and sequential. H&E staining section was the last wax slice. Lung tissue infected with *Cryptococcus* was used as a positive control, and normal human lung tissue was used as a negative control.

Routine H&E staining: the sections were stained using an H&E staining kit (Baso Diagnostics Inc., Zhuhai, China). Paraffin sections were dewaxed with xylene and gradient alcohol, and then stained with H&E. Finally, the sections were dehydrated in a gradient and sealed with a neutral resin.

Grocott methenamine silver staining: sections were stained using a GMS staining kit (Baso Diagnostics Inc., Zhuhai, China). Paraffin sections were dewaxed with xylene and gradient alcohol and then oxidized in 8% chromic acid solution for 20 min. After pretreatment with 0.5% sodium metabisulfite solution, the sections were immersed in a methenamine-silver nitrate solution preheated to 60°C and placed in an electro-thermostatic blast oven at 60°C for 40 min. The sections were subsequently washed with distilled water, treated with a 5% sodium thiosulfate solution, and counterstained with a light green solution. Finally, the sections were dehydrated in a gradient and sealed with a neutral resin.

Periodic acid-Schiff staining: sections were stained using a PAS staining kit (Baso Diagnostics Inc., Zhuhai, China). Paraffin sections were deparaffinized using xylene and gradient alcohol. After oxidation with a 1% periodic acid solution for 10 min, they were treated with Schiff reagent in the dark for 20 min. The sections were then washed under running water for 5 min and counterstained with Mayer’s hematoxylin. Finally, the sections were dehydrated in a gradient and sealed with a neutral resin.

Alcian blue staining: sections were stained using an AB staining kit (Baso Diagnostics Inc., Zhuhai, China). Paraffin sections were deparaffinized using xylene and gradient alcohol. After staining with AB (pH 2.5) solution for 20 min, the sections were stained with nuclear fast red solution for 5 min. Finally, the sections were dehydrated in a gradient and sealed with a neutral resin.

### Staining Evaluation and Statistical Analysis

*Cryptococcus*-positive diagnosis with GMS, PAS, and AB staining was indicated by brown-black staining with a light green background, magenta staining with a blue nucleus, and blue staining with a red nucleus, respectively. Pathological diagnoses of all stained sections and cases were independently evaluated by two pathologists; any discrepancies were resolved by a third senior doctor. Statistical analysis was performed using SPSS software (version 25.0; SPSS, Chicago, IL, United States). The quantitative index of normal distribution was described by x̄ ± s. The classification index was expressed by case count and percentage. McNemar’s test was used to compare the sensitivity of GMS, PAS, and AB stains. *P*-values of <0.05 were considered statistically significant.

## Results

### Pathological Characteristics of Study Cohort

A total of 152 (53 women; average age, 52 years; age range, 23–81 years) patients with PC were included. Among them, 102 (67.1%) patients had no clinical symptoms and were admitted because of the presence of pulmonary nodules on physical examination. Leukocyte count, neutrophil percentage, and C-reactive protein levels were within the normal ranges in 141 (92.8%), 135 (88.8%), and 140 (92.1%) patients, respectively. This evidence suggests that in cases diagnosed with PC, infection indicators may not show a correlation, which may be related to the body’s immunity. The imaging findings of patients with PC were not specific, and multiple nodules (96 cases, 63.2%) were common, with spiculation and lobulation of suspected lung cancer in 67 (44.1%) and 78 (51.3%) cases, respectively. These findings suggest a lack of imaging or clinical presentation specificity associated with PC (Table 1).

**TABLE 1:** Demographic and clinical characteristics of 152 patients with pulmonary cryptococcosis.

Characteristic	Study cohort *n* = 152
Age (years)	51.09 ± 11.84
Sex	
Male	99 (65.1%)
Female	53 (34.9%)
Symptom	
No symptoms	102 (67.1%)
Cough	31 (20.4%)
Expectoration	17 (11.2%)
Chest pain	18 (11.8%)
Back pain	7 (4.6%)
Chest distress	4 (2.6%)
Fever	1 (0.7%)
Diseases history	
Healthy	50 (32.9%)
Hypertension	30 (19.7%)
Diabetes mellitus	14 (9.2%)
Hepatitis B	8 (5.3%)
Tuberculosis	3 (2.0%)
Connective tissue disease	2 (1.3%)
Laboratory tests	
Leukocyte count, ×10^9^/L	
Normal ranges (3.5–9.5)	141 (92.8%)
>9.5	7 (4.6%)
<3.5	4(2.6%)
N%	
Normal ranges (40–75)	135 (88.8%)
>75	10 (6.6%)
<40	7 (4.6%)
CRP, mg/L	
Normal ranges (0–8.2)	140 (92.1%)
>8.2	12 (7.9%)
Nodule multiplicity	
Size (cm)	1.65 ± 0.93
Solitary nodule	56 (36.8%)
Multiple nodules	96 (63.2%)
Spiculation	67 (44.1%)
Lobulation	78 (51.3%)
Necrosis	
Yes	39 (25.7%)
No	113(74.3%)

*N%, percent of neutrophil; CRP, C-reactive protein. Normal ranges: laboratory clinical reference value range.*

### Grocott Methenamine Silver Staining for Detecting Pulmonary Cryptococcosis

Statistical analysis showed that the detection rates of GMS, PAS, and AB staining were 100% (152/152), 94.7% (144/152), and 81.6% (124/152), respectively, in paraffin-embedded tissue sections from 152 patients with PC. The sensitivity of GMS staining was 100%, whereas that of PAS staining was 94.7%, and the consistency between them was 94.7%; McNemar’s test showed that the difference between the two stains was statistically significant (*P* = 0.008). The sensitivity of AB staining was 81.6%, and the consistency between GMS and AB was 81.6%; there was a significant difference between the two methods (*P* < 0.001). The sensitivity of PAS was higher than that of AB, and the consistency between the two methods was 84.2%; the difference between the two methods was statistically significant (*P* < 0.001). These findings demonstrated that the sensitivity of GMS staining was significantly higher than those of PAS staining (*P* = 0.008) and AB staining (*P* < 0.001) (Table 2).

**TABLE 2:** Sensitivity values of the three staining methods for pulmonary cryptococcosis.

	Positive cases	Total	Sensitivity (%)	Consistency (%)	*P*-value^a^
**GMS vs PAS**				94.7	0.008
GMS	152	152	100		
PAS	144	152	94.7		
**GMS vs AB**				81.6	<0.001
GMS	152	152	100		
AB	124	152	81.6		
**PAS vs AB**				84.2	<0.001
PAS	144	152	94.7		
AB	124	152	81.6		

*^a^McNemar’s test. GMS, Grocott methenamine silver; PAS, periodic acid-Schiff; AB, Alcian blue.*

### Staining Quality, Stability, and Identifiability

**FIGURE 1:**
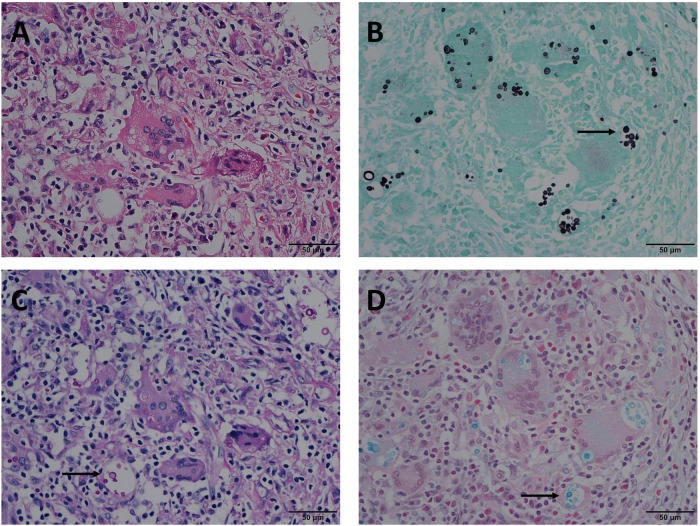
Mode A: GMS(+), PAS(+), AB(+) in the same view of lung tissues with pulmonary cryptococcosis (male, aged 42 years, multiple nodules, maximum diameter of 1 cm). **(A)** H&E staining reveals morphological characteristics of granulomatous inflammation, and circular structures resembling *Cryptococcus* spores. **(B)** GMS(+) reveals *Cryptococcus* spores (arrow) that are clear, brown-black, round, or oval structures, with different sizes and clear morphological characteristics such as capsule and narrow-based budding against the bright green and clean background. **(C)** PAS(+) reveals *Cryptococcus* spores (arrow) stained magenta of varied intensity, including some very lightly stained structures. **(D)** AB(+) reveals *Cryptococcus* spores (arrow) stained blue of varied intensity. GMS, Grocott methenamine silver; PAS, periodic acid-Schiff; AB, Alcian blue; H&E, hematoxylin and eosin. (Original magnifications: **A–D**, ×400).

The positivity rates of GMS, PAS, and AB staining were mainly observed in four modes (Table 3). The most common mode was positive for GMS, PAS, and AB staining. There were 122 cases in Mode A, accounting for 80.3% of the study cohort. In these cases, H&E staining revealed morphological features of granulomatous inflammation in the lung tissue. Hyperplastic epithelioid cells and multinucleate giant cells were observed, with diaphanous round or oval structures in the cytoplasm, suspected to be *Cryptococcus* spores (Figure 1A), and infiltration of interstitial lymphocytes and plasma cells. Occasionally, necrosis was observed in the lung tissue. GMS staining revealed a brown-black color that contrasted with a bright green background. *Cryptococcus* presented clearly as round or oval structures with different sizes. It had a thick mucinous capsule and narrow-based budding (Figure 1B). The background was clean, and *Cryptococcus* could be quickly recognized at low- or medium-power magnification. Meanwhile, PAS and AB stained *Cryptococcus* with magenta and blue of varied intensity, respectively; some spores stained normal magenta and blue, and some were lightly stained (Figures 1C,D). Moreover, under the same field of view, PAS and AB staining showed lower *Cryptococcus* density than did GMS staining.

**TABLE 3:** Positive rates of the three staining methods for pulmonary cryptococcosis.

	GMS stain (%)	PAS stain (%)	AB stain (%)	Number
Model A	+	+	+	122
Model B	+	+	−	22
Model C	+	−	+	2
Model D	+	−	−	6
Total	152 (100)	144 (94.7)	124 (81.6)	152

*GMS, Grocott methenamine silver; PAS, periodic acid-Schiff; AB, Alcian blue.*

**FIGURE 2:**
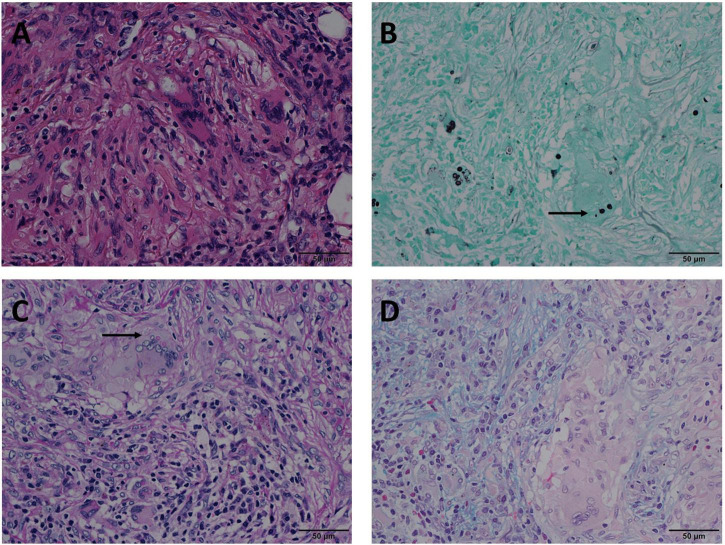
Mode B: GMS(+), PAS(+), AB(–) in the same view of lung tissues with pulmonary cryptococcosis (male, aged 66 years, solitary nodule, maximum diameter of 0.8 cm). **(A)** H&E staining reveals circular structures resembling *Cryptococcus* spores. **(B)** GMS(+) shows brown-black *Cryptococcus* spores (arrow) that are clear and intuitive. **(C)** PAS(+) shows *Cryptococcus* spores (arrow) stained magenta and non-specific staining interference of neutral mucosubstances and glycogen. **(D)** AB(–) shows no obvious *Cryptococcus* blue but reveals non-specific staining comparable to the Cryptococcal structure. GMS, Grocott methenamine silver; PAS, periodic acid-Schiff; AB, Alcian blue; H&E, hematoxylin and eosin. (Original magnifications: **A–D**, ×400).

**FIGURE 3:**
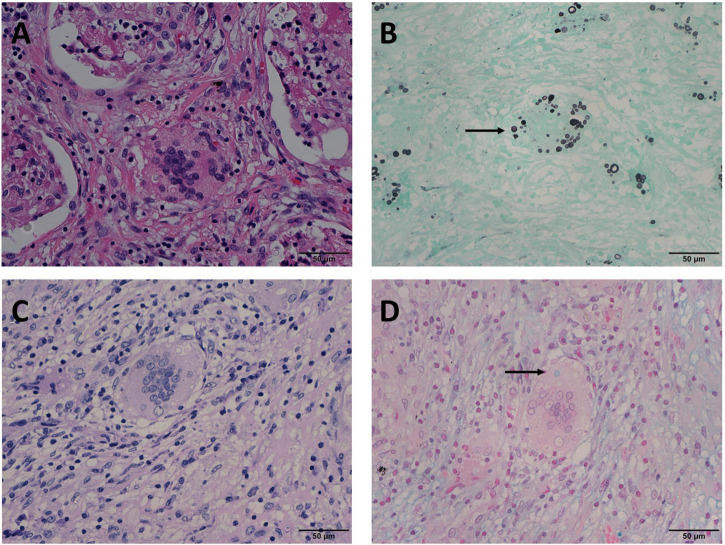
Mode C: GMS(+), PAS(–), AB(+) in the same view of lung tissues with pulmonary cryptococcosis (female, aged 56 years, multiple nodules, maximum diameter of 1.4 cm). **(A)** H&E staining reveals circular structures resembling *Cryptococcus* spores. **(B)** GMS(+) highlights brown-black *Cryptococcus* spores (arrow). **(C)** PAS(–) shows no obvious magenta *Cryptococcus* but reveals colorless or reddish round-like structures. **(D)** AB(+) reveals *Cryptococcus* blue (arrow) within the multinucleated giant cells, with interfering blue acid mucosubstances. GMS, Grocott methenamine silver; PAS, periodic acid-Schiff; AB, Alcian blue; H&E, hematoxylin and eosin. (Original magnifications: **A–D**, ×400).

**FIGURE 4:**
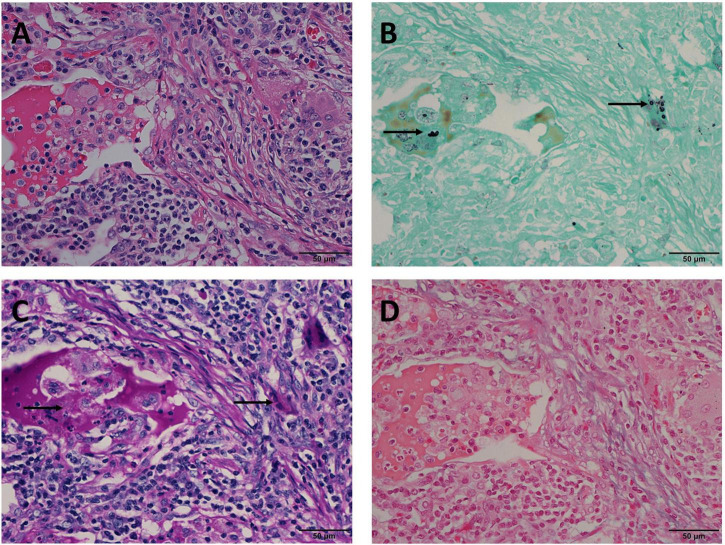
Mode D: GMS(+), PAS(–), AB(–) in the same view of lung tissues with pulmonary cryptococcosis (male, aged 61 years, solitary nodule, maximum diameter of 1.2 cm). **(A)** H&E staining shows circular structures resembling *Cryptococcus* spores. **(B)** GMS(+) reveals *Cryptococcus* spores (arrows) that are observable by their brown-black color. **(C)** PAS(–) does not reveal *Cryptococcus* magenta, but suspicious magenta round-like structures are covered with non-specific staining (arrows). **(D)** AB(–) does not reveal *Cryptococcus* blue. GMS, Grocott methenamine silver; PAS, periodic acid-Schiff; AB, Alcian blue; H&E, hematoxylin and eosin. (Original magnifications: **A–D**, ×400).

In Mode B, 22 (14.5%) cases were positive for GMS and PAS staining, and negative for AB staining. In these 22 cases, H & E staining revealed circular structures resembling *Cryptococcus spores* (Figure 2A), and, *Cryptococcus* was clearly highlighted in brown-black by GMS staining with a clean background (Figure 2B). Although *Cryptococcus* can be found in some multinucleated giant cells, PAS staining requires careful identification of magenta circular structures with obvious positive characteristics under high-power magnification, which is time-consuming and laborious (Figure 2C). It was difficult to identify the morphology of blue *Cryptococcus* with AB staining at low- or high-power magnification. Although there were similar structures to *Cryptococcus*, the final diagnosis involved non-specific staining after careful identification (Figure 2D). In contrast, there were 2 (1.3%) cases in which GMS and AB staining were positive and PAS was negative in Mode C. H&E staining revealed circular structures resembling *Cryptococcus spores* (Figure 3A), and GMS staining highlighted brown-black Cryptococcus spores (Figure 3B). The circular structures suspected to be *Cryptococcus* in the PAS staining sections of the two cases were colorless or reddish, with no obvious magenta circular structures (Figure 3C). AB staining revealed *Cryptococcus* blue within the multinucleated giant cells, with interfering blue acid mucosubstances (Figure 3D). In Mode D, 6 (3.9%) cases were positively diagnosed with GMS staining only. In this type of cases, H&E staining and GMS staining tissue morphology were similar to corresponding Mode A, Mode B and Mode C (Figures 4A,B). The magenta circular structure of the suspected *Cryptococcus* with PAS staining was covered with non-specific staining, making it difficult to identify magenta *Cryptococcus* (Figure 4C). AB staining did not reveal Cryptococcus blue (Figure 4D). These results indicated that GMS staining was superior to PAS and AB staining in terms of quality, stability, and identifiability.

### Grocott Methenamine Silver Was Optimal for Diagnosing Pulmonary Cryptococcosis

**FIGURE 5:**
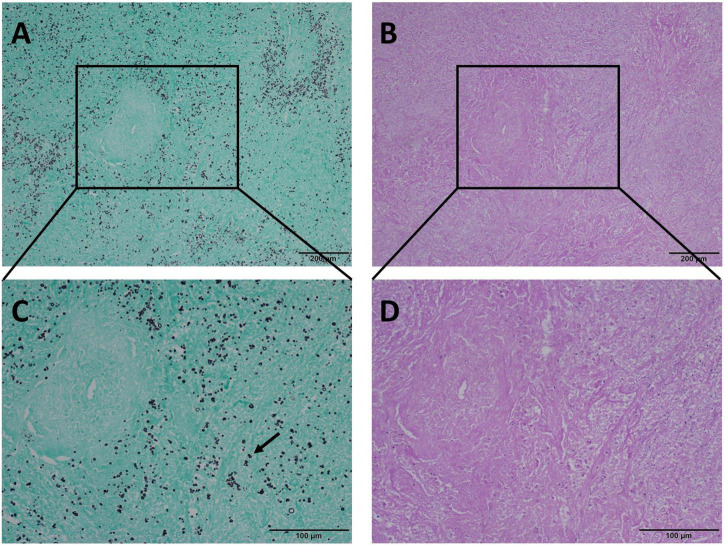
Comparison of GMS and PAS staining in the same view of pulmonary necrotic tissues with pulmonary cryptococcosis. **(A,C)** *Cryptococcus* spores are clearly visible at low and medium-power magnification, and the morphological characteristics of narrow-based budding (arrow) are clear with GMS staining at medium-power magnification. **(B)** With PAS staining, the necrotic area is purplish-red, and *Cryptococcus* is not visible at low-power magnification. **(D)** PAS staining shows the necrotic area as purplish-red color, and *Cryptococcus* is not easily distinguished at medium-power magnification. GMS, Grocott methenamine silver; PAS, periodic acid-Schiff. (Original magnifications: **A,B**, ×100; **C,D**, ×200).

In PC sections stained with GMS, *Cryptococcus* was brown-black and prominent against the bright green clean background. The size, structure, and narrow-based budding were observed at low- or medium-power magnification (Figures 5A,C). However, there was interference of red coloration revealing neutral mucosubstances, glycogen, and some connective fiber tissues in PAS staining. Specifically, when *Cryptococcus* accumulated in the necrotic foci, magenta *Cryptococcus* was similar in color to the necrotic foci and was not easily distinguished (Figures 5B,D). However, when the staining background had a large number of normal human cells, it interfered with *Cryptococcus* observation, which usually requires high magnification. Similarly, AB staining interfered with the blue acid mucosubstances. Non-specific PAS and AB staining resulted in a structural pattern resembling that of *Cryptococcus*. In PAS staining, this structural pattern was red, round-like, with a colorless middle and a reddish periphery (Figure 2C), whereas for AB staining, the same structural pattern was formed with a colorless middle and blue outer ring (Figure 2D). This structural pattern, comparable to that of *Cryptococcus*, can be missed even at high-power magnification. Comparative analysis showed that the identifiability of GMS was significantly better than those of PAS and AB staining, and that GMS was optimal for diagnosing PC.

## Discussion

At present, pathological examination of lung biopsy tissues, which can directly display *Cryptococcus*, is the gold standard for the diagnosis of PC. According to the consensus definitions of invasive fungal diseases from the European Organization for Research and Treatment of Cancer and Mycoses Study Group Education and Research Consortium (EORTC/MSG), PC can be diagnosed histopathologically (Donnelly et al., 2020). However, various histological staining methods have been developed. In clinical pathological practice, most pathologists adopt a panel (GMS, PAS, and AB) as the selection method; however, no consensus has been reached on the suitability of this panel.

*Cryptococcus* is a narrow-based budding yeast, round or ovoid, with large size differences and a diameter of 2–20 μm; it is surrounded by a wide mucopolysaccharide capsule (Guarner and Brandt, 2011; Roden and Schuetz, 2017). *Cryptococcus* is not revealed by H&E staining, and GMS, PAS, and AB staining are often used to assist in diagnosis. In this study, we retrospectively analyzed paraffin-embedded tissue sections from 152 patients with PC to evaluate the sensitivity of these three staining methods for the diagnosis of PC. The results showed that 152 (100.0%), 144 (94.7%), and 124 (81.6%) patients were GMS(+), PAS(+) and AB(+), respectively, indicating that the sensitivity of GMS was significantly higher than that of PAS (*P* = 0.008) and AB (*P* < 0.001) staining.

In our study, GMS staining showed excellent sensitivity (100%) in 152 PC tissues, which was significantly better than that of PAS staining (94.7%, *P* = 0.008). Both techniques rely on the use of oxidizing agents (chromic or periodic acid) to create aldehyde-binding sites for Schiff reagents or silver ions. Chromic acid is a strong oxidizing agent that oxidizes the 1,2-ethanediol hydroxyl group on the sugar moiety into an aldehyde group (–CHO) and ultimately into a carboxyl group (–COOH) (Shiogama et al., 2015). The strength of the oxidizing agent, incubation time, and density of the polysaccharide in the relevant tissue structure determine the course of the reaction. Because fungal cell walls are rich in polysaccharides, the hydroxyl groups on these sugar groups are not completely transformed into carboxyl groups within a certain period, but many oxidation processes remain at the aldehyde group stage. Other structures with low polysaccharide content, such as the basement membrane and reticular fibers, are completely converted to carboxyl groups. Therefore, GMS staining is highly identifiable, revealing the fungal cell wall as brown-black, while other histological structures are barely visible. In contrast, due to the “weak” oxidation of periodic acid, PAS staining can only oxidize hydroxyl groups to aldehyde groups (Jackson, 2011). It results in a large number of positive tissues and cells (Layton and Bancroft, 2019), which explains the defects of the principle of PAS staining of fungi; the present findings are consistent with this staining principle.

In the sections stained with PAS, magenta glycogen, neutral mucosubstances, and other substances were observed in the tissues of most cases. These non-specific staining substances make it difficult to identify *Cryptococcus* against the background of many red stains, increasing the risk of misdiagnosis and missed diagnosis. The sections showed diffuse magenta non-specific staining of the necrotic site, comparable to that of *Cryptococcus*, after the use of the Schiff reagent; *Cryptococcus* was difficult to recognize in cases where *Cryptococcus* was present at the necrotic sites. In particular, when there is little fungi in the sections of PC cases, missed diagnosis is more likely. The PC sections of GMS (+) and PAS (−) cases revealed a single or several *Cryptococcus* that were dispersed. Li et al. (2021) reported a case of pulmonary cryptococcal infection in a patient with normal immune function and a small amount of fungi with positive GMS and negative PAS staining. In contrast, GMS staining with bright green counterstaining clearly revealed brown-black *Cryptococcus* against the green background. In most cases, *Cryptococcus* could be observed at low-power magnification, making diagnosis easier and more efficient, and suggesting that GMS is superior to PAS in the diagnosis of *Cryptococcus*.

In AB staining, AB is a copper phthalein cyanine dye, which is blue because of the presence of copper in the molecule. AB is a positively charged fluoride salt that can combine with negatively charged acidic groups in acid mucosubstances in tissues to produce blue hues (Fagan et al., 2020). With AB staining, the capsule of *Cryptococcus* containing acid mucosubstances becomes blue. AB staining (pH 2.5) can help in the diagnosis of *Cryptococcus* because AB only reacts with *C. neoformans* (capsule) and *Blastomyces dermatitidis* (wall) (Lazcano et al., 1993). In our study, the sensitivity of AB staining was only 81.6%, which was lower than those of GMS and PAS (both *P* < 0.001); this finding may be accounted for by the absence of the *Cryptococcus* capsule. In addition, when the lung tissue secretes more acid mucosubstances, it makes the acidic mucus wrap into a round-like structure. This structure is comparable to that of *Cryptococcus* blue and is difficult to identify. It can affect that accuracy of interpretation and delay section reading. In a panel (GMS, PAS, and AB) test to diagnose PC, GMS clearly reveals *Cryptococcus* against a contrasting background. However, PAS and AB have many interfering factors, which will bring more unnecessary trouble to clinicopathological histological diagnosis and increase unnecessary medical expenses for patients. The present findings suggest that GMS may be the most effective approach to PC diagnosis. When pulmonary cryptococcal infection is suspected, GMS staining is recommended for accurate and cost-effective diagnosis.

## Conclusion

In summary, GMS is superior to PAS and AB staining in the diagnosis of pulmonary fungi. GMS staining has better sensitivity and identifiability, clear contrast, and a clean background, with less interference from various factors. It can be easily interpreted by clinical pathological diagnosticians and has high discrimination accuracy. Therefore, GMS is optimal for the histological diagnosis of PC.

## Data Availability Statement

All datasets used in this study are publicly available on the Research Data Deposit public platform (www.researchdata.org.cn). The RDD number of this article is RDDA2021443948.

## Ethics Statement

The studies involving human participants were reviewed and approved by the Institutional Review Board of Sun Yat-sen University Cancer Center (B2021-452-01). All samples were anonymous and the requirement of obtaining informed consent was waived by the Institutional Review Board of Sun Yat-sen University Cancer Center.

## Author Contributions

JC designed this study and revised the manuscript. SW and JL analyzed the data and wrote the manuscript. RW and LZ collected the data. MH, YX, and XZ assisted in analyzing the data of PC patients. All authors approved the final version for submission.

## Conflict of Interest

The authors declare that the research was conducted in the absence of any commercial or financial relationships that could be construed as a potential conflict of interest.

## Publisher’s Note

All claims expressed in this article are solely those of the authors and do not necessarily represent those of their affiliated organizations, or those of the publisher, the editors and the reviewers. Any product that may be evaluated in this article, or claim that may be made by its manufacturer, is not guaranteed or endorsed by the publisher.
